# Supplementary material for: Lipopolysaccharide inhalation recruits monocytes and dendritic cell subsets to the alveolar airspace
Source: Nat Commun. 2019 Apr 30;10:1999. doi: 10.1038/s41467-019-09913-4 (PMC6491485; doi:10.1038/s41467-019-09913-4)
Supplement: Supplementary file 1 — Supplementary Information [file 41467_2019_9913_MOESM1_ESM.pdf]

Lipopolysaccharide inhalation recruits monocytes and dendritic cell subsets to the alveolar airspace

Jardine and Wiscombe et al

Supplementary Figure 1

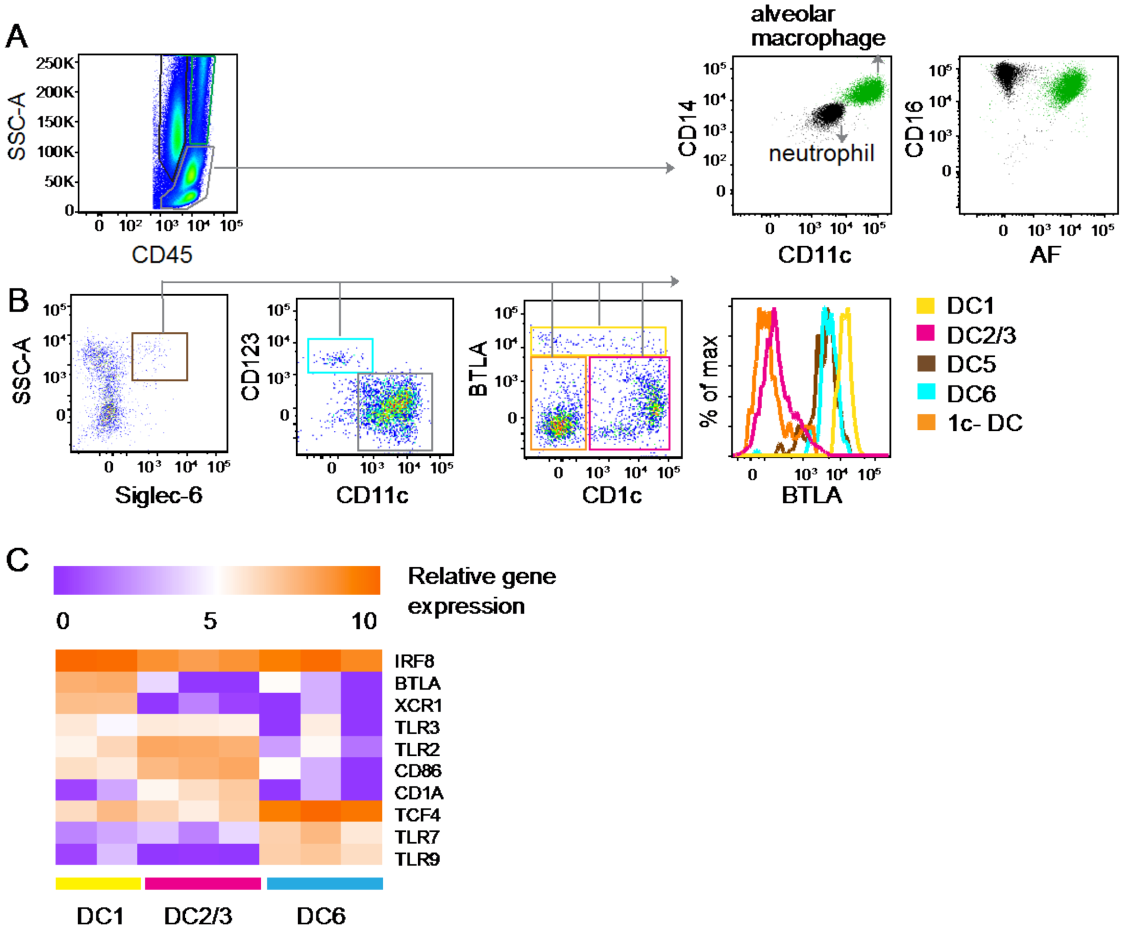

Supplementary Figure 1: Flow cytometry analysis of MP subsets in BAL

Additional detail on surface antigen expression by MP subsets, as defined by the gating strategy shown in Figure 1, with the relevant plots recreated here.

A) Demonstration that alveolar macrophages are $CD14^{+}CD11c^{+}CD16^{+}$ cells with autofluorescence in the FITC channel and neutrophils are $CD14^{lo}CD11c^{lo}CD16^{+}$ cells without autofluorescent properties.

B) BTLA expression on DC subsets.

C) Verification that the gating strategy identifies DC populations with expected gene expression profiles. Selected gene expression by DC1, DC2 and pDC sorted from BAL and measured on NanoString array.

Supplementary Table 1: Participant characteristics

	LPS	Saline
Age (years)<br>Mean (SD)	21 (1.83)	21 (1.39)
Gender<br>Male; n (%)<br>Female; n (%)	 7 (70)<br>3 (30)	 4 (44)<br>5 (56)
Ethnicity<br>Caucasian; n (%)<br>Other; n (%)	 8 (80)<br>2 (20)	 8 (89)<br>1 (11)
Body surface area (m^2)<br>Mean (SD)	1.90 (0.19)	1.74 (0.16)
Forced vital capacity (L)<br>Mean (SD)	4.39 (0.73)	3.93 (1.02)
Forced vital capacity (% predicted)<br>Mean (SD)	99.86 (7.58)	98.98 (15.55)
Forced expiratory volume in 1 second (L)<br>Mean (SD)	4.39 (0.72)	3.93 (1.02)
Forced expiratory volume in 1 second (% predicted)<br>Mean (SD)	100.85 (9.82)	98.48 (14.13)

Supplementary Figure 2

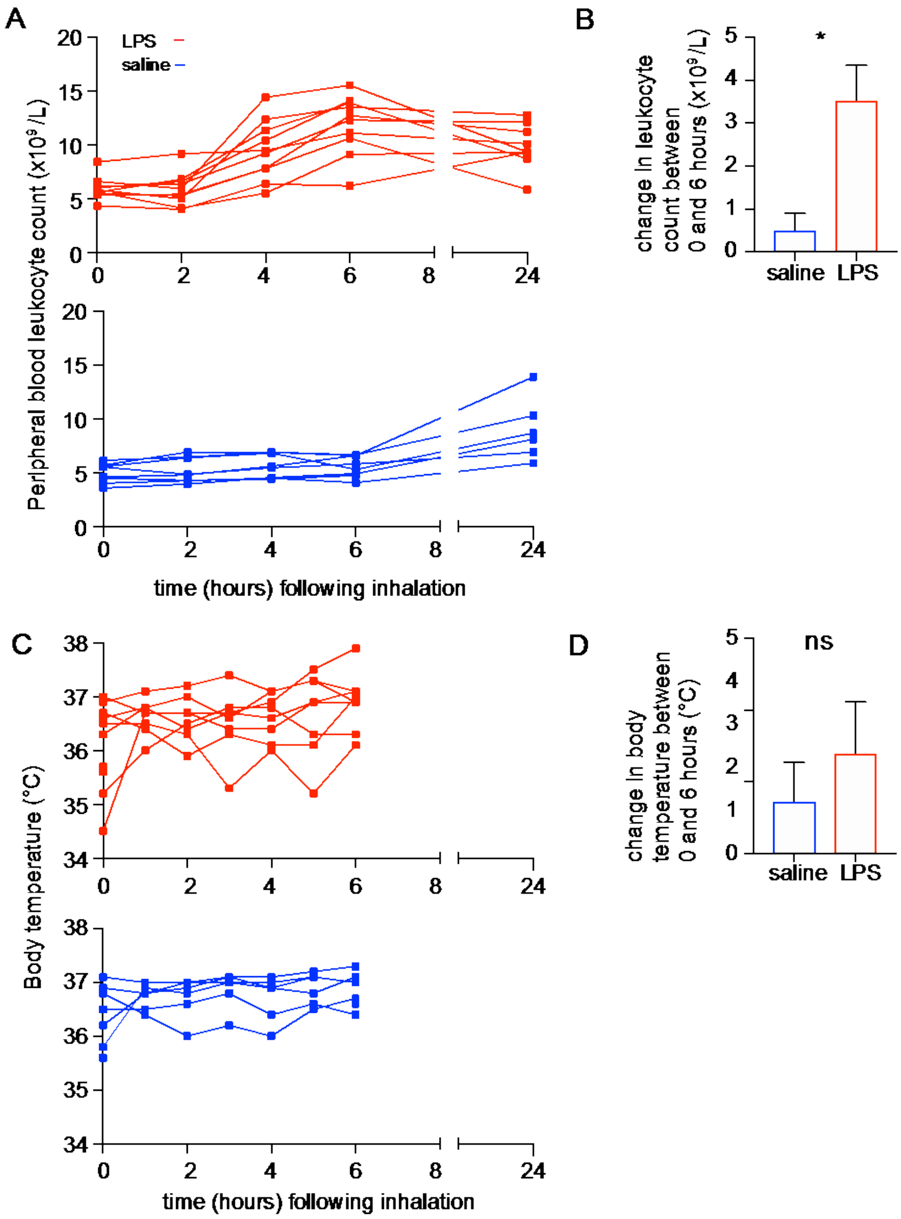

Supplementary Figure 2: Clinical parameters following inhalation of LPS or saline

A Peripheral blood leukocyte count 2, 4, 6 and 24 hours following inhalation of LPS or saline. One participant receiving saline had difficult venous access and no samples were obtained. 24-hour blood samples were omitted in 2 saline and 1 LPS recipients due to volunteer preference.
B) Change in leukocyte count between 0 and 6 hours in each recipient. Bars show mean change and error bars show SEM. * $p<0.05$ by unpaired t-test.
C) Body temperature measured hourly until 6 hours following inhalation of LPS or saline. Data sets are incomplete in 3 saline and 3 LPS recipients.
D) Change in leukocyte count between 0 and 6 hours in each recipient. Bars show mean change and error bars SEM. 'ns'= $p>0.05$ by unpaired t-test.

Supplementary Figure 3

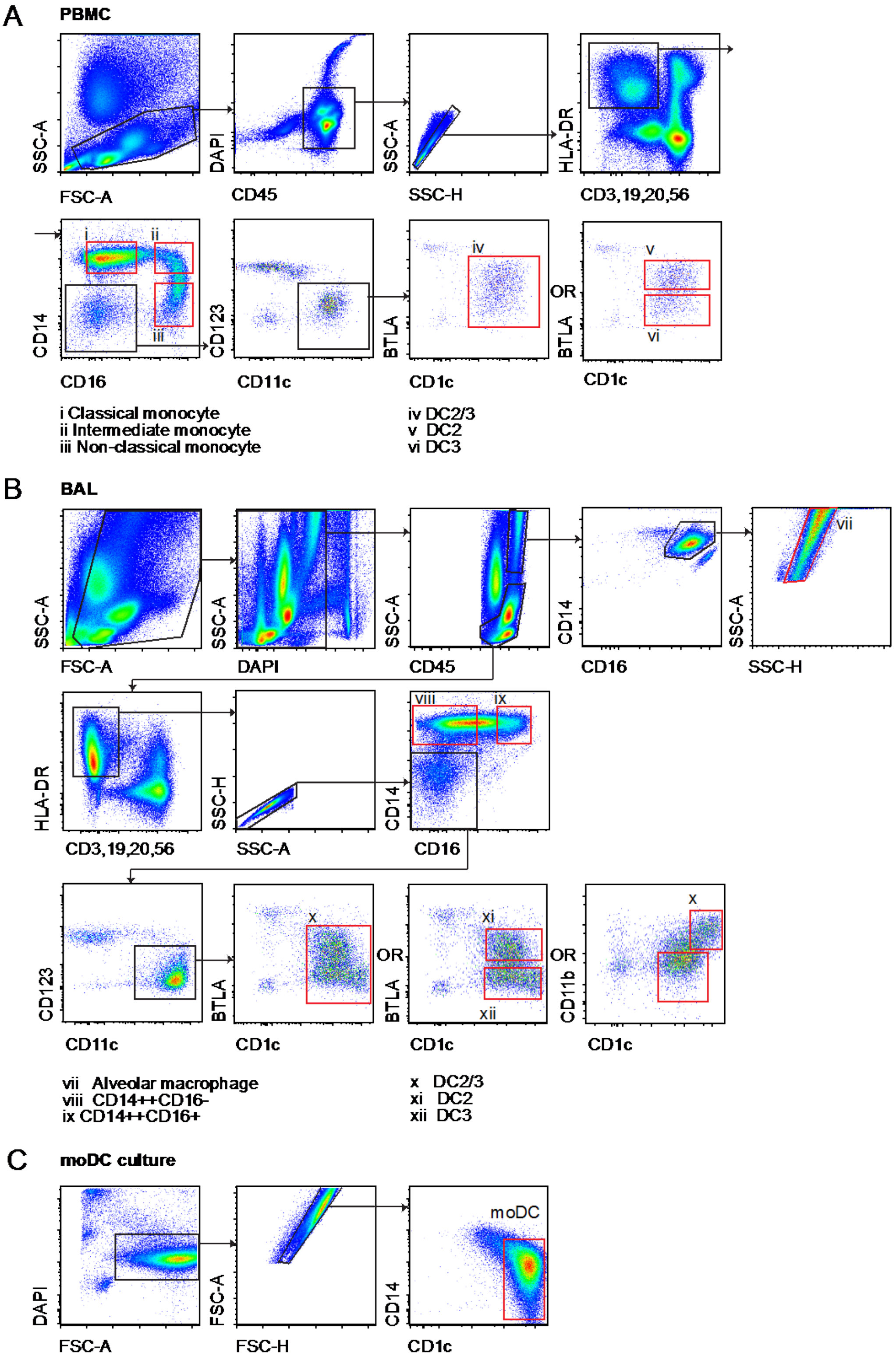

Supplementary Figure 3: Gating strategies used for cell sorting

A) Gating strategy to sort monocytes and DCs from PBMC for NanoString expression analysis. Monocyte data are presented in Fig. 2A,B,C and Fig. 3B. Classical monocytes were also used for *in vitro* generation of moDC. DC data are presented in Fig. 3B,D and Fig. 4A,C.

B) Gating strategy to sort monocyte/macrophages and DCs from BAL for NanoString expression analysis and *in vitro* assays. LPS-BAL CD1c^{+} DCs were sorted one of three ways: as total DC2/3 (gate xi), split into BTLA^{hi} DC2 and BTLA^{lo} DC3 (gates xii and xiii) or split into CD1c^{hi} and CD1c^{lo} fractions (gates xiv and xv). Early analysis revealed that sorted CD1c^{hi} and CD1c^{lo} fractions had homogeneous gene expression, so data were subsequently considered technical replicates of DC2/3. Monocyte/macrophage NanoString data are presented in Fig. 2A,B,C,D and Fig. 3B. Alveolar macrophage NanoString data are presented in Fig 5A. DC2/3 NanoString data are presented in Fig. 3B,D,E and Fig. 4A,C. DC1 and DC6 NanoString data are included in Supplementary Figure 1. Data from *in vitro* co-culture of sorted cells and T cells are presented in Fig. 3C and Fig 4D. Data from *in vitro* stimulation of sorted cells are presented in Fig. 5.D

C) Gating strategy to sort moDC from culture for NanoString expression analysis, presented in Fig. 3B.
