# Supplementary material for: Lipopolysaccharide inhalation recruits monocytes and dendritic cell subsets to the alveolar airspace
Source: Nat Commun. 2019 Apr 30;10:1999. doi: 10.1038/s41467-019-09913-4 (PMC6491485; doi:10.1038/s41467-019-09913-4)
Supplement: Supplementary file 3 — Description of Additional Supplementary Files [file 41467_2019_9913_MOESM3_ESM.pdf]

Description of Additional Supplementary Files

File Name: Supplementary Data 1
Description: **Differentially expressed genes.** Details of differential gene expression between LPS BAL $CD14^{++}CD16^{+}$ MP and SS BAL $CD14^{++}CD16^{+}$ MP and HC blood classical monocytes (**DEGs Fig 2C,D**); LPS BAL DC2/3 and SS BAL DC2/3 and HC blood DC2/3 (**DEGs Fig 3D,E**); LPS BAL AM and SS BAL AM (**DEGs Fig 5A**). Details of genes used to discriminate monocyte/macrophages from DCs (**Gene set Fig 3B**).
